# Efficient FPGA Implementation of Automatic Nuclei Detection in Histopathology Images

**DOI:** 10.3390/jimaging5010021

**Published:** 2019-01-17

**Authors:** Haonan Zhou, Raju Machupalli, Mrinal Mandal

**Affiliations:** Department of Electrical and Computer Engineering, University of Alberta, Edmonton, AB T6G 2R3, Canada

**Keywords:** FPGA implementation, hardware architecture, image processing, histopathology, generalized Laplacian of Gaussian filter, nuclei detection, mean Shift clustering

## Abstract

Accurate and efficient detection of cell nuclei is an important step towards the development of a pathology-based Computer Aided Diagnosis. Generally, high-resolution histopathology images are very large, in the order of billion pixels, therefore nuclei detection is a highly compute intensive task, and software implementation requires a significant amount of processing time. To assist the doctors in real time, special hardware accelerators, which can reduce the processing time, are required. In this paper, we propose a Field Programmable Gate Array (FPGA) implementation of automated nuclei detection algorithm using generalized Laplacian of Gaussian filters. The experimental results show that the implemented architecture has the potential to provide a significant improvement in processing time without losing detection accuracy.

## 1. Introduction

**Figure 1:**
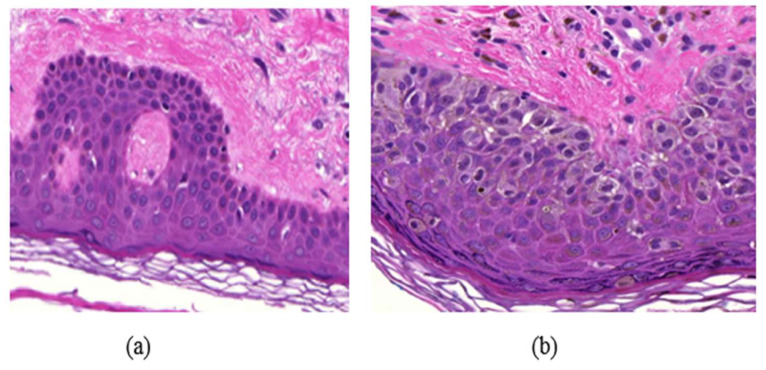
Example of a skin Whole Slide image (WSI), (**a**) normal skin image, (**b**) melanoma affected skin image.

Many diseases are diagnosed based on the cellular structures in their respective tissue specimens as the cellular structures can provide quantitative information about the diseases and help in the study of disease progression. For example, the density of cell nuclei in histological images is an important feature for automatic breast or skin tumor grading [1]. The difference between normal skin cells and abnormal skin cells can be seen in Figure 1. In human intervened diagnosis procedure, histopathologists typically examine the tissue under a microscope, and the diagnostic accuracy depends on the pathologists’ personal experience, which sometimes leads to intra and inter observer variability [2]. To overcome these limitations, several computer-aided diagnosis (CAD) techniques have been proposed in the literature for the diagnosis. Due to a wide variety of nuclei appearances in different organs, and staining procedures, accurate and efficient segmentation of cell nuclei is an important step in most histopathology-based CAD techniques. The detection of cells in a histology image may also be the first step towards cell segmentation.

Since cell nuclei typically have circular shapes, they can be considered as blob-like structures which can be detected efficiently using scale-space theory. Xu et al. [1] proposed an efficient technique for nuclei detection using directional gLOG (generalized Laplacian of Gaussian) kernels on red channel image of H&E (Hematoxylin and Eosin) strained color histopathology images. The technique generates intermediate response maps using directional gLOG kernels. It is possible to obtain more than one point from different response maps, corresponding to the same nuclei in the input image. Therefore, seeds from these response maps are merged using mean-shift clustering. It gives a promising performance in nuclei seeds detection.

The histological images typically have a large size. For example, a 20 mm^2^ glass slide tissue scanned with a resolution of 0.11625 μm/pixel (at 40× magnification) will consist of about 2.96 × 10^10^ pixels, and will approximately require 80 GB of storage space in an uncompressed color format (24bits/pixel) [2]. In addition, gLoG kernels generally require significant computation. As a result, the nuclei detection techniques typically have high computational complexity, and software implementation on general purpose processors (GPP) requires a significant amount of processing time. For real-time diagnosis, it would be helpful to develop a hardware accelerator for faster nuclei detection. With advances in CMOS and fabrication technology, Field Programmable Gate Array (FPGA) and Graphical Processing Unit (GPU) are being widely used as a High-Performance Computing (HPC) solution to overcome the GPP limitations. GPUs are efficient for data parallel applications with high memory bandwidth requirement and are typically programmed using high-level languages, such as CUDA. On the other hand, FPGAs have more flexibility than GPUs and are efficient for both *data* and *task* parallel applications.

In this paper, we propose an FPGA-based hardware architecture for cell nuclei detection in a histology image obtained using H&E stain. The proposed architecture uses data parallelism. To reduce the computation burden and power consumption, floating point arithmetic is implemented in a fixed-point form without losing much accuracy. The architecture has low latency. The organization of the rest of the paper is as follows. Section 2 gives details on nuclei detection algorithm and its implementation. Section 3 presents experimental results and performance evaluation. Discussion on results is presented in Section 4, followed by conclusion in Section 5.

## 2. Materials and Methods

**Figure 2:**
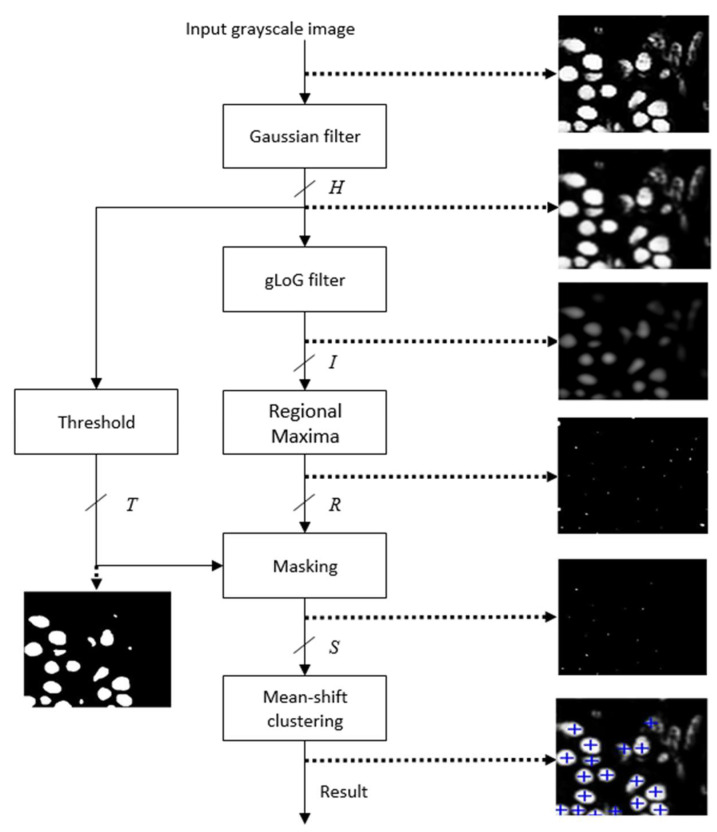
Schematic of the proposed nuclei detection technique.

The schematic of the proposed accelerator architecture for nuclei detection is shown in Figure 2. It has been found that [1] the nuclei can be detected efficiently using the red channel of the H&E stained RGB image. Therefore, the red channel of the histology image is used as the input gray scale image. The architecture mainly contains six modules: Gaussian filter, gLoG filter, Regional Maxima, Thresholding, Masking and Mean-shift clustering. The Gaussian filter smooths an input image. The gLoG filter is then applied to generate response maps corresponding to different scales and orientation of the gLoG kernels. The Regional Maxima module generates nuclei seed candidates from the response maps. In order to reduce the number of false positive seeds, a mask is generated by applying the Thresholding module on the Gaussian filter output and Masking is done on the nuclei seed candidates generated from Regional Maxima module. Finally, the Mean-shift clustering module clusters the remaining seed candidates to obtain coordinates of different nuclei centers. The anticipated results of different modules are also shown in Figure 2. Implementation details of each module is given in the following sections.

### 2.1. Gaussian Filter

**Figure 3:**
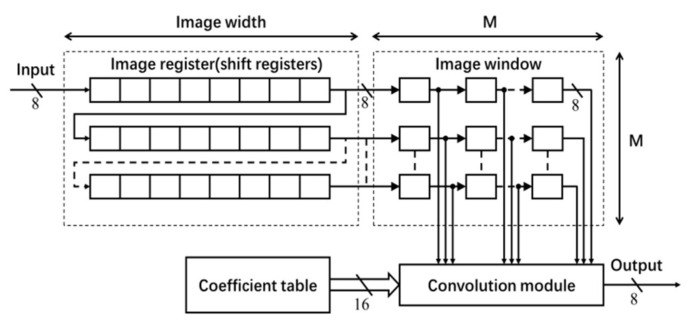
Architecture of 2-D Gaussian Filter.

The architecture for the Gaussian filter is shown in Figure 3, which mainly consists of a coefficient table, an image register unit, an image window and a convolution module [3]. The coefficients of the M×M Gaussian filter are generated offline. The normalized filter coefficients (in floating-point data type) are converted into fixed-point data type. In this implementation, 16-bit fixed-point representation (with fraction length of 14) has been used (without any significant loss of accuracy). The filter coefficients are stored in ROM IP core on the FPGA board.

To enable the process of shifting the window of 2-D filter coefficients for a raster scan of the entire image, *M* shift register IP cores of length equal to the image width (see Figure 3) are used for generating a Serial-In-Parallel-Output (SIPO) image register unit [3]. Each shift register stores one row of image data. Input to this register unit is an 8-bit image pixel data comes at a rate of one pixel per clock. The *M* pixels from each shift register are transferred to the image window to access randomly in convolution.

**Figure 4:**
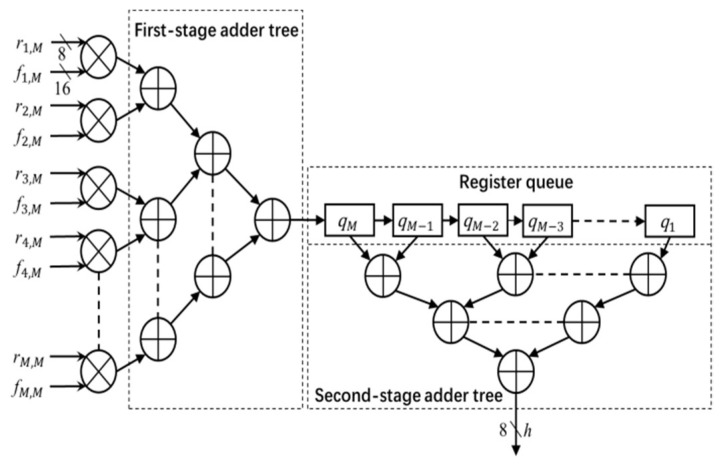
Convolution module. Multiplication of data and coefficients are shown for *M*th column.

The architecture of the convolution module is shown in Figure 4. The module uses the image data *r* (stored in the image window) and filter coefficients *f* (stored in the coefficient table) to calculate the output *h*. The entire convolution process with *M* × *M* size filter is divided into *M* cycles. In each cycle, one column (e.g., *i*th column) of the image window data *r* and coefficient table data *f* pass through the multiplier array and first stage adder tree, and is then stored in the register queue. In each subsequent cycle, the register data shifts right by one unit, and the next columns of image data and filter coefficients go through the module and the output is stored in the register queue. This process continues for *M* times. After *M* cycles, the outputs of every column (stored in register queue) are added by the second stage adder to calculate the convolution output *h* (pixel value in the Gaussian filter output image *H*). In this work, *h* is truncated into 8-bit precision and the output image *H* is stored in the FPGA block RAM.

### 2.2. 2-D gLoG Filter

Because the cell nuclei in digital histopathological images typically have circular or elliptical shapes, the 2-D gLoG filters are used for nuclei detection [1]. The nuclei are detected by convolving the image *H* with a 2-D gLoG filter. The gLoG filters are generated from a bank of gLoG kernels $\nabla^{2}G(x,y)$ as defined below [1]:

$$\nabla^{2}G(x,y)=\frac{\partial^{2}G(x,y)}{\partial x^{2}}+\frac{\partial^{2}G(x,y)}{\partial y^{2}},$$

where G(x,y) is a 2-D Gaussian function defined as follows.

$$G(x,y)=\lambda \cdot e^{-(ax^{2}+2bxy+cy^{2})}$$

**Figure 5:**
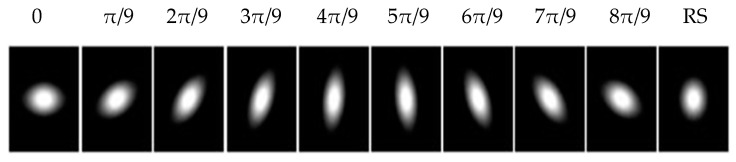
Ten 2-D gLoG filters with different orientations.

Note that *a*, *b* and *c* are functions of scale $\left(\sigma_{x},\sigma_{y}\right)$ and orientation *θ* of the Gaussian kernels [1,4]. By changing the scales and the orientation, a set of gLoG kernels can be obtained. In this paper, we generate gLoG kernels $\left(\sigma_{x},\sigma_{y}\right)$ with $\sigma_{x}>\sigma_{y}$ ranging from 6 to 12 insteps of 0.5 and nine orientations θ,{θ=nπ/9,n=0,1,...,8}. The nine gLoG filters corresponding to nine orientations are generated by adding up gLoG kernels of the same orientation, but with different scales. Special kernels, whose $\sigma_{x}=\sigma_{y}$ are rotational symmetric and their structures are independent of the orientation, are summed separately to form a rotationally symmetric gLoG filter. In this paper, 10 gLoG filters are used (see Figure 5), with nine filters of different orientations and one rotationally symmetric (RS) filter. A total of 10 response maps, with one response map from each gLoG filter, are generated.

For hardware implementation, the architecture of the gLoG filter module is similar to that of the Gaussian filter described in the previous section, except the filter size and coefficients. In this work, the size of the gLoG filter is set to 25 × 25 in order to match the size of typical nuclei in the input data. As the gLoG filter coefficients are independent of the image data, they are calculated offline, converted into 16-bit precision (with 14-bit fractional value), and stored in ROM IP cores on the FPGA. The output response map (denoted by *I*) from each gLoG filter is stored with 8-bit precision in the block RAM on the FPGA.

### 2.3. Regional Maxima Calculation

Regional maxima are connected components of pixels with a constant grayscale value, *t*, whose external boundary pixels all have a value less than *t* [5,6]. As the regional maxima in a gLoG filter response map *I* are usually around the nuclei centers, they are detected in this module and considered as candidate pixels to calculate the nuclei centers.

**Figure 6:**
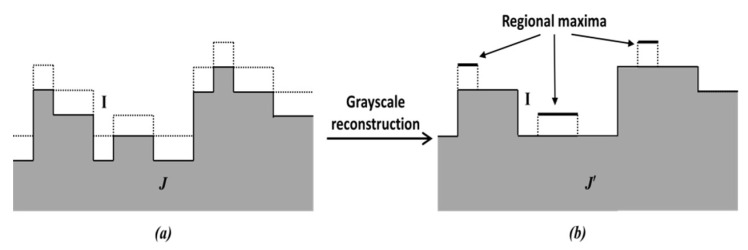
Principle of regional maxima calculation. (**a**) dotted lines, *I*, shows Mask, shaded portion shows Marker *J*, (**b**) *J’* indicates the reconstruction of *J.*

The principle of regional maxima calculation used in this paper is shown in Figure 6. In Figure 6a, the response map *I* (denoted by dotted lines) is used as the mask image. A marker image J=I−1 (shown by full lines) is generated and stored in an FPGA block RAM (if *J* < 0, it is set to 0). A *hybrid grayscale reconstruction* algorithm [6], described below, is then performed on the marker image *J*, and let the output be denoted by $J^{\prime }$. After that, $I-J^{\prime }$ is calculated, and where the outcome value is 1, the corresponding pixel is considered as the regional maxima. This is illustrated in Figure 6b.

**Figure 7:**
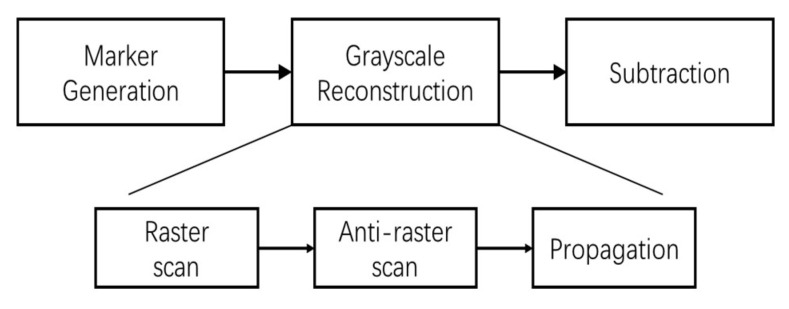
Block diagram of regional maxima calculation.

Figure 7 shows the block schematic of the *Regional Maxima* module, which has 3 parts: *Marker generation*, *Grayscale Reconstruction* and *Subtraction*. Function of *Marker Generation* (J=I−1) and *Subtraction* $\left(I-J^{\prime }\right)$ parts are mentioned in the previous paragraph. The *Grayscale Reconstruction* of the marker image *J* is done in 3 steps, *Raster scan*, *Anti-raster scan* and *Propagation*, which are explained in the following.

After generating the mask *I* and the marker *J*, a raster scan of these two images is performed. Let *p* denote pointer of the current pixel in the scanning, and *q* denote its neighbor’s pixel positions.

**Figure 8:**
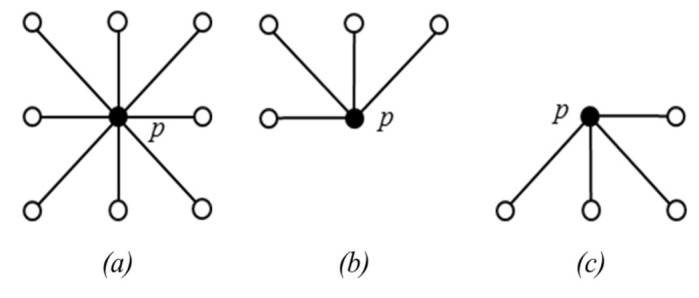
Illustration of neighbors of pixel *p*. (**a**) N(p). (**b**) $N^{+}(p)$, (**c**) $N^{-}(p)$.

The eight neighbors of *p* are denoted as N(p) (see Figure 8a). The 4 neighbors reached before *p* in a scan order are denoted as $N^{+}(p)$ (see Figure 8b). The maximum value of $\left\{J(p),J(q),\;q\in N^{+}(p)\right\}$ is then calculated and denoted as *s*. Finally, the J(p) is updated with min{s,I(p)}. After the raster scan, an anti-raster scan (scanning from the bottom pixel) of *I* (i.e., the original image) and updated *J* is performed in a similar way. This time, it checks if for a pixel *p*, there exists a pixel $q\;(q\in N^{+}(p)$ such that J(q)<J(p) and J(q)<I(q), the *q* value is stored in the FIFO (First In First Out) queue.

After the anti-raster scan, the propagation step is performed on the FIFO structure. In the beginning, the FIFO is checked, if it is empty, the process of grayscale reconstruction is completed; if it is not, the point which is at the beginning of the FIFO is popped out and denoted as *p*. The values of I(p),J(p),J(q) and I(q), q∈N(p) are read from images *I* and *J*. If there exist any q∈N(p), such that J(q)<J(p) and J(q)≠I(q), the minimum value between J(p) and I(q) is given to J(q) and the *q* is put into the queue. Then another round of the loop begins. This process continues until there are no data in the queue. The updated *J* is the grayscale reconstructed marker image and is denoted as $J^{\prime }$.

Finally, a binary response map $R=I-J^{\prime }$ is calculated for each gLoG output, and stored in the FPGA RAM, where a binary value of 1 indicates the regional maxima.

### 2.4. Thresholding

The thresholding module converts the Gaussian lowpass filtered image into a binaryimage of foreground (i.e., nuclei) and background pixels. The threshold value for an input image can be calculated using any adaptive threshold methods for more effectiveness in eliminating false regional maxima in binary response map *R*. However, in this implementation, a global threshold value is used for simplicity. The thresholded image *T* is generated from the lowpass filtered image *H* as follows:

$$T(m,n)=\{\begin{array}{cc} 1\;(nuclei) & if\;H(m,n)<\tau \\ 0\;(backg) & otherwise \end{array},$$

where (*m,n*) is a pixel coordinate. The threshold value τ is calculated offline using Otsu’s method. Implementation of above the Equation is done using a comparator, and *T* is stored in FPGA RAM.

### 2.5. Masking

A response map $R_{i},\;1\leq i\leq 10$ corresponding to 10 response maps may have false regional maxima, due to noise in the input image. The *masking* module eliminates the false maxima that are located outside nuclei masks *T* generated by the thresholding module. The output *M* of the module is calculated as follows:

$$M_{i}(m,n)=R_{i}(m,n)\;\&\;T(m,n),$$

where & is a logical AND operation. The pixel locations with $M_{i}(m,n)=1$ correspond to nuclei seed candidates. The seed candidate locations from all *M* matrices are combined and stored in the FPGA RAM. Let the set of candidate nuclei coordinates be denoted by *S*.

### 2.6. Mean-Shift Clustering

For one nuclear region, there can be more than one candidate nuclei in S [1]. As the candidates corresponding to a nucleus are geometrically close, they can be clustered to obtain one center for each nucleus. In this paper, the nuclei candidates are clustered using a mean-shift (MS) clustering algorithm [7], and center for each nucleus is obtained by calculating the mean coordinate of members of the corresponding cluster.

**Figure 9:**
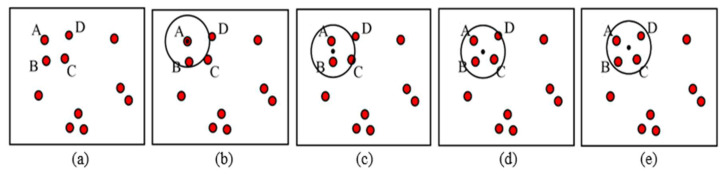
Mean shift clustering principles, (**a**) candidates in a binary image, (**b**) Kernel centered at A, (**c**) Kernel centered at mean of A and B, (**d**) kernel centered at mean of A, B and C, (**e**) kernel centered at mean of A, B, C and D. Finally, A, B, C and D clustered into one nucleus.

**Figure 10:**
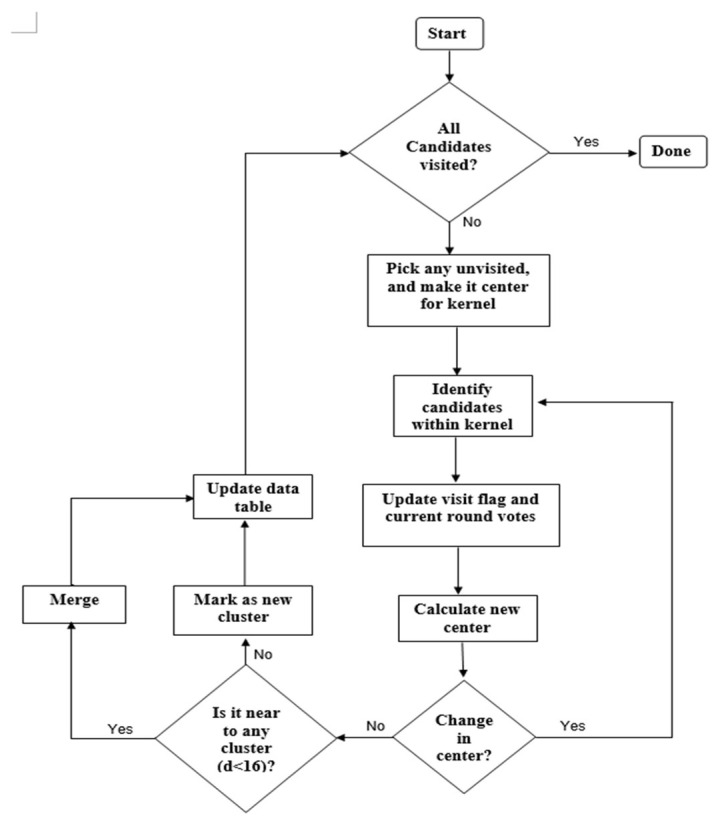
Flowchart of implemented mean-shift (MS) clustering.

The MS clustering is like a hill climbing algorithm which involves shifting a certain type of kernel iteratively to a higher density region until convergence. This is illustrated in Figure 9, where the nuclei candidates are shown with red dots. To start the algorithm, pick any unvisited candidate, let it be A and place the kernel center at A. Check if any other candidates are within the kernel (of radius *r*). In this example, candidate B is within the kernel (see Figure 9b). Calculate the mean of A, B and shift the kernel center to mean position (see Figure 9c). Now re-check if any new candidate is included within the kernel. Figure 9c shows that candidate C is within the kernel. The mean of {A, B, C} is calculated, and the kernel is shifted to the new mean position. The iteration continues until the kernel is settled and no new candidate is included. After convergence, all candidates within the kernel are clustered and the center of the kernel is considered as the nucleus for that cluster. The MS clustering then picks up another unvisited candidate and generates a cluster in a similar manner. The process is continued until all the candidates are clustered. The overall flowchart of the MS clustering is shown in Figure 10.

**Figure 11:**
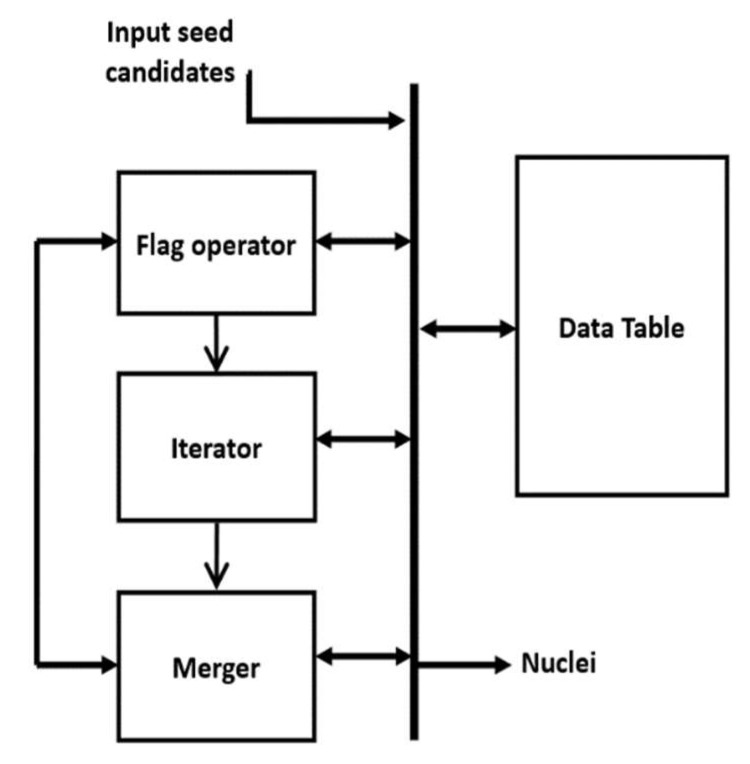
Architecture of MS clustering.

In this paper, the MS kernel is defined as a circle with radius *r* = 8 pixels. The implemented architecture of the MS clustering is shown in Figure 11. The architecture has four modules: *Flag operator*, *Iterator*, *Merger* and *Data table*. The *Data table* structure is shown in Table 1, which stores the seed candidates, four intermediate parameters for each candidate (*visit-flag, current-round votes, maximum votes and cluster number*) and *identified Nuclei*. *Visit-Flag* identifies candidates that are visited in the clustering process (flag is set to 1 for visited candidates and 0 for unvisited candidates). *Current-round votes* $V_{c}$ indicate the number of iterations done in current clustering when a candidate is within the kernel geometry. $V_{c}$ is set to zero at the beginning of each cluster generation. *Maximum votes* $V_{M}$ store the maximum value of *current-round votes* a candidate has achieved in all previous cluster generation and the *cluster number* (*L*) denotes the cluster that has obtained maximum votes for a seed candidate. The table entries are updated at the end of each cluster generation. *Identified nuclei* (*N*) stores the center of each converged cluster.

In the beginning, the flag operator checks the *visit-flags* table. If there are any unvisited candidates (whose visit-flag is 0), it picks one of those unvisited candidates randomly (*S*_i_) and gives it to the *iterator*. The *iterator* places the kernel center at candidate *S*_i_ and finds all the candidates within the kernel geometry (i.e., distance < r). For those candidates (within the kernel), *visit flag* is set to 1 and *current-round vote* value is increased by 1. The iteration is repeated with the center of kernel shifted to mean position of candidates within the kernel. The process repeats until kernel center is converged (i.e., no change in mean position). The final converged point $C_{k}$ is then sent to the *Merger* module.

The *Merger* module scans through the *Identified nuclei* column. If there exists a previously generated Nuclei $C_{l}(l\neq k)$ whose distance to the current convergent point $C_{k}$ is smaller than a threshold (e.g., 16 pixels), then $C_{k}$ should merge with $C_{l}$. The value for the merged nuclei $C_{l}$ is changed to the mean coordinate of $C_{l}$ and $C_{k}$. In *cluster number* column, if $L(s_{i})=l$, the *maximum votes* value for corresponding candidates are changed to $V_{M}(S_{i})=V_{M}(S_{i})+V_{C}(S_{i})$. If there are no *Identified nuclei* within the threshold distance to $C_{k}$, then $C_{k}$ becomes a new Nuclei and added to *Identified nuclei* column. Finally, the comparison between *current round votes* and *maximum votes* are done. For a candidate $S_{i}\in C_{k}$ if $V_{M}(S_{i})<V_{C}(S_{i})$ then $V_{M}(S_{i})$ is changed to $V_{C}(S_{i})$ and $L(S_{i})$ to *k* (indicating that candidate *S_i_* belongs to the new cluster $C_{k}$). Example data format can be seen in Table 1.

After the operations in the *Merger* module finished, the *Flag operator* module scans through the *Visited-flags table* to check whether there are any unvisited candidates. If there are unvisited candidates, the *Iterator* is enabled again to generate a new cluster, otherwise, the *Clustering* module is disabled, and the MS clustering is done. The mean coordinate of the candidates belonging to one cluster (corresponding to a nucleus) is considered as the seed coordinate of the detected nuclei.

## 3. Results

**Figure 12:**
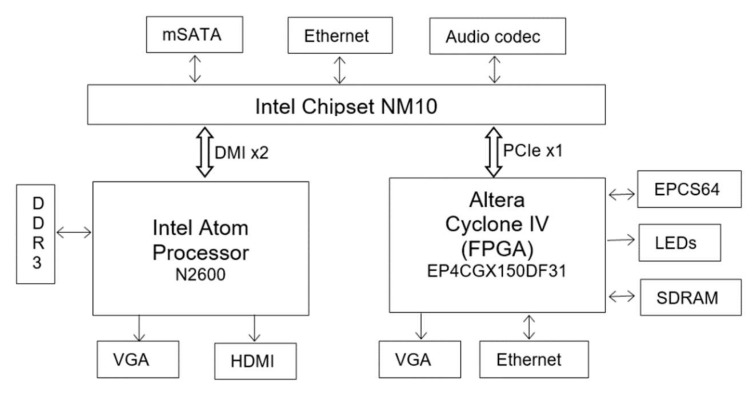
Simplified schematic of DE2i-150 FPGA development board Architecture (refer to [8] for more detailed schematic).

The proposed architecture is implemented on DE2i-150 FPGA development board, developed by Terasic [8]. A simplified schematic of the DE2i-150 development board is shown in Figure 12 (refer to the Terasic site [8] for complete details). The board is an embedded platform with Intel N2600 Atom Dual core processor (Intel Corporation, Santa Clara, CA, USA) [9] coupled with Altera’s Cyclone IV GX FPGA (Altera (Acquire by Intel), Santa Clara, CA, USA). The Atom processor has 64-bit Instruction set, 1M cache running at 1.6 GHz clock speed, and is connected to external DDR3 memory. The Atom pairs with Intel® NM10 Express Chipset through Direct Media Interface (DMI) to provide rich I/O capabilities and flexibility via high-bandwidth interfaces, such as PCI Express, Serial ATA (SATA), mSATA, and Ethernet. Cyclone IV FPGA is connected to Atom through PCI Express (PCIe) bus and NM10. The FPGA is connected to 128MB SDRAM (32 bits width), 4MB SSRAM and 64MB Flash memory with 16-bit mode. Both Atom and FPGA has a VGA connector to interface with monitor. In this paper, the proposed architecture is implemented using only the FPGA (Atom processor is not used).

In the implementation, the block RAMs on FPGA are used to store intermediate results instead of available 128 MB SDRAM. Because, this SDRAM has a latency of four clock cycles, with a maximum allowed clock frequency of 100 MHz, which can degrade the proposed architectures performance. Because there is a lot of intermediate date generated and it should be accessible randomly. But implementing the same architectures (with block RAM replaced by available memory) on high-end FPGA boards having lower latency and higher clock frequency for memory can give a similar performance with fewer resources.

### Parameter Configuration

A few parameters for the proposed architecture must be defined before generating bit file for the hardware [1]. The parameters are application dataset dependent. The Gaussian filter size *M* depends on the amount of noise in the input image and minimum nuclei size to detect. Filter size cannot be more than the size of smaller nuclei expect to detect, otherwise it blurs the nucleus. The rest of the parameters, like gLoG filter size, threshold value, MS clustering bandwidth are to be set according to possible nuclei size range in application dataset. In this experiment, the parameters are not fine-tuned to a dataset, but for the same parameter configuration the proposed hardware should give similar results with software (MATLAB) implementation. To check the proposed hardware flexibility with parameters across a different dataset, experiments are done on two sets of parameters. Parameter configuration of two sets are shown in Table 2. The hardware provided similar results as MATLAB [10] with respect to each set of parameters.

**Figure 13:**
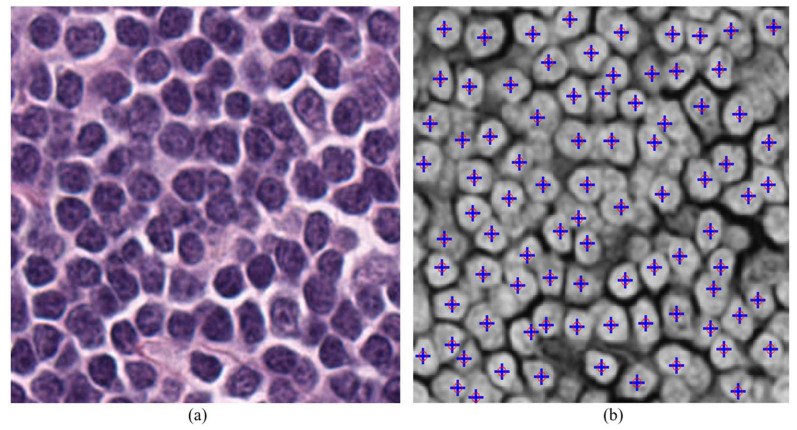
Results of Nuclei detection with set 1 parameters configuration on both MATLAB and hardware, (**a**) input color image, (**b**) output detected nuclei indicating on complement red channel input image, blue color ‘+’ indicates the nuclei detected using hardware, red circle ‘o’ indicates the nuclei detected using MATLAB.

The architecture performance is evaluated by comparing its execution time and accuracy with MATLAB for 256 × 256 size images. Before generating the bit-file for the hardware, the parameters have to be configured according to application and input image data should be initialized into ROM IP block using .hex/.mif file format. As the input is red channel data (complemented) of H&E stained images, MATLAB is used to generate the .hex/.mif file with red channel data. The resources utilized by the architecture for 256 × 256 size image on the FPGA (for set-1 parameters configuration) is presented in Table 3. MATLAB is running on AMD Athlon II CPU (Advanced Micro Devices, Inc., Austin, TX, USA) at 2.90 GHz with 4GB RAM. To compare the results, the detected nuclei coordinates in the FPGA are read out and marked on the MATLAB results. Figure 13 shows the nuclei detection results using both hardware and MATLAB.

The execution time on FPGA measured using counter register, it increments for every millisecond (i.e., the execution time is in millisecond precision) and final execution time is displayed through available 15 LEDs on the board. The average execution time over 10 different input images on both hardware and MATLAB are presented in Table 4.

The accurate detection of nuclei in the input image depends on the parameters mentioned above. Therefore, in this paper the accuracy of proposed hardware evaluated with respect to the results from the MATLAB (software) version (2017b, MathWorks, Natick, MA, USA) [10]. It is observed in Figure 13 that both versions give similar results for the same parameters configuration. Finding the optimized parameters to compare the accuracy of detection is beyond the scope of this paper as this paper is focused more on FPGA implementation.

## 4. Discussion

The proposed architecture has been implemented and tested for a small image patch of size 256 × 256. But the architecture can easily be extended to larger image size, as histopathology images are typically very large in size. The total processing time for a full resolution image, with a size 20,000 × 20,000, is expected to be in the order of hours in a regular CPU. With optimized implementation and FPGA boards with higher clock frequency, the overall processing time is expected to be in the order of minutes.

The proposed architecture shows a modest 34.5% performance improvement compared to a regular CPU, which is mainly because of the lower clock frequency (100 MHz) of the FPGA board. The speed-up factor can be improved further by exploiting the data and task parallelism in each sub module. Empirically, it can be said that smaller modules implemented on FPGA give low performance (speed up) improvement over the software (on general purpose processor) implementation as the data parallelism achieved with FPGA can be overshadowed by operating clock frequency. For larger modules, having possible data parallelism can give significant performance improvement worth of going for special hardware accelerator. Our larger goal is to design a CAD system for histopathology for which the nuclei detection is one module. It is expected that the other modules in the CAD system will have larger speed up resulting in a high overall system performance.

## 5. Conclusions

A software implementation of the CAD technique requires a significant amount of processing time. To assist the pathologists in real time, this processing time should be reduced. In this paper, an FPGA based hardware accelerator for the Nuclei detection has been proposed, and its performance is evaluated by implementing it on DE2i-150 FPGA development board. The hardware accelerator shows a significant performance improvement over a MATLAB, even though it is running at a lower clock frequency. Further performance improvement can be achieved by exploring the data and task parallelism exists in the algorithm. Once the nuclei are detected on the histopathology images, next step in the CAD process is to segment the nuclei and perform the diagnosis in real-time. It is the base model to develop a complete CAD accelerator for many diagnosis processes (processing of large histopathology images).

## Figures and Tables

**Table 1:** Illustration of data table format. Table entry numbers are randomly filled.

Seed Candidates	Visit-Flag	Current Round Votes (*V_C_*)	Maximum Votes (*V_M_*)	Cluster Number (*L*)	Identified Nuclei (*C*)
(x1, y1)	1	2	2	1	(a1, b1)
(x2, y2)	1	2	3	2	(a2, b2)
(x3, y3)	0	0	0	.	(a3, b3)

**Table 2:** Configured parameters table.

Parameter	Set 1	Set 2
Gaussian filter size	7 × 7	8 × 8
gLoG size	25 × 25	49 × 49
Threshold	155	150
MS bandwidth	8	6

**Table 3:** Resource utilization table.

Resources	Utilized
Total Logical elements	34,475
Memory Bits	4,711,338
PLLs	1
Embedded Multipliers	70

**Table 4:** Performance comparison.

Platform	Clock Frequency	Execution Time (in sec)
MATLAB (on CPU)	2.90 GHz	1.694
Proposed implementation	100 MHz	1.108

## References

[1] Xu H., Lu C., Berendt R., Jha N., Mandal M. (2017). Automatic Nuclei Detection based on Generalized Laplacian of Gaussian Filters. IEEE J. Biomed. Health Inf..

[2] Lu C., Mandal M. (2015). Automated analysis and diagnosis of skin melanoma on whole slide histopathological images. Pattern Recognit..

[3] Al-Jobouri L. (2017). Design of a Convolutional Two-Dimensional Filter in FPGA for Image Processing Applications. Computers.

[4] Kong H., Akakin H.C., Sarma S.E. (2013). A generalized Laplacian of Gaussian filter for blob detection and its applications. IEEE Trans. Cybern..

[5] Soile P. (1999). Morphological Image Analysis: Principles and Applications.

[6] Vincent L. (1993). Morphological Grayscale Reconstruction in Image Analysis: Applications and Efficient Algorithms. IEEE Trans. Image Process..

[7] Comaniciu D., Meer P. (2002). Mean shift: A robust approach toward feature space analysis. IEEE Trans. Pattern Anal. Mach. Intell..

[8] DE2i-150 FPGA Development Board Specifications and Architecture. https://www.terasic.com.tw/.

[9] Intel Corporation. https://www.intel.com/content/www/us/en/homepage.html.

[10] Mathworks, Inc.. https://www.mathworks.com/.

